# Lock-Down Effect on the Mental Health Status of Healthcare Workers During COVID-19 Pandemic

**DOI:** 10.3389/fpsyt.2021.683603

**Published:** 2021-08-13

**Authors:** Weam Fageera, Fawzi Babtain, Ahmad S. Alzahrani, Hussain M. Khrad

**Affiliations:** ^1^Research Center, King Faisal Specialist Hospital and Research Center, Jeddah, Saudi Arabia; ^2^Neuroscience Department, King Faisal Specialist Hospital and Research Center, Jeddah, Saudi Arabia; ^3^College of Medicine, Alfaisal University, Riyadh, Saudi Arabia

**Keywords:** COVID-19, anxiety, depression, healthcare workers, Saudi Arabia

## Abstract

**Background:** The psychological impact that outbreaks and pandemics could inflict on healthcare workers has been widely studied; yet, little is known about the impact of the lockdown measures.

**Objectives:** To assess the magnitude of depression and anxiety among healthcare professionals before and after lifting of the lockdown restrictions in Saudi Arabia.

**Methods:** Surveys targeting healthcare workers were circulated twice: during the lockdown, and 8 weeks after lifting of lockdown. Anxiety and depressive symptoms were assessed using Generalized Anxiety Disorder (GAD-7) and Patient Health Questionnaire-9 (PHQ-9) scales.

**Results:** A total of 947 healthcare workers, with the mean age of (37 ± 8.9) responded to the surveys. Among these, 23–27% respondents reported clinically significant levels of anxiety and depression. Whereas, easing of the lockdown restrictions was shown to be associated with decreasing mean scores of PHQ-9 and GAD-7. The noted burden fell heavily on female workers, those with a current or a history of psychiatric disorders, suffering from chronic diseases, being in workplaces with high exposure to COVID-19 or in contact with COVID-19 patients, nurses, as well as those who were living with elderly and perceived their physical and mental health as “much worse” compared to the time before the pandemic.

**Conclusion:** Our findings identified several predictors for anxiety and depression at different time-points of the pandemic. Thus, priority to psychological support measures might be needed for these groups.

## Introduction

Living in the midst of one of the major public health crises has fundamentally impacted various aspects of people's lives. The novel coronavirus disease (COVID-19) was first reported to the World Health Organization (WHO) Country Office in China in December 2019 (1). Few months later, in March 2020, the WHO declared COVID-19 a pandemic (2). In Saudi Arabia, the first case of COVID-19 was confirmed on March 2, 2020. Due to the high contagiousness, rapid spread of the virus, severity of illness presentation, and lack of effective vaccine, the government of Saudi Arabia had taken rigorous unprecedented precautionary measures to curb the spread of the disease, including declaring a national level “lockdown.” On March 23, 2020, the government of Saudi Arabia enforced local lockdown for 21 days, which was later extended up to 91 days. Lockdown measures included the mandatory closure of schools, travel restrictions, limiting the movement within and between all regions of the Kingdom, suspending commercial activities, self-quarantining, and implementing partial and a full-day curfew (3). As of June 21, 2020, the lockdown was completely lifted across the Kingdom (Supplementary Figure 1).

Despite the success of this action on containing the spread of the disease, it could have had a major impact on the mental health and well-being of people. Several studies were conducted concerning the psychological impact of COVID-19 in Saudi Arabia, most of them were carried out during the time of the lockdown. Majority of these studies indicated a moderate to severe psychological impact (4–7). These findings, in a way, echo those from previous studies conducted in different parts of the world (8–10). Furthermore, the psychological impact of COVID-19 was also investigated in different sub-populations, including healthcare workers. Since, the healthcare workers were facing a huge burden from the beginning of this crisis, which was emphasized by the WHO calling for actions to prevent serious consequences (11). In addition to stress of higher risk exposure due to the direct/indirect contact with infected patients, healthcare workers were also experiencing the restricted lockdown measures, social isolation, disrupted normal life activities, media information overload and panic. All these factors could be overwhelming, leading to a wide spectrum of serious psychological consequences. Several studies revealed that the healthcare workers could have a higher tendency to develop psychological problems compared to the general population (5, 7, 12, 13). Most of these studies were conducted during the lockdown period in many countries; yet, little is known about the impact of the lockdown and the temporal distribution of mental state of healthcare workers.

This study is the first to assess the mental health outcomes and associated risk factors in healthcare workers at different stages of the pandemic in Saudi Arabia. Herein, we sought to assess the levels of anxiety and depression among healthcare workers during the time of the lockdown and after returning to “normalcy” in Saudi Arabia and to identify the factors that are associated with the worsening of these symptoms.

## Methods

### Ethics Statement

The study was approved by the local Research Advisory Committee and Research Ethics Committee of King Faisal Specialist Hospital and Research Centre-Jeddah (KFSHRC-J).

### Study Design, Participants, and Procedure

This is a cross-sectional, survey-based study. Healthcare workers who were working in Saudi Arabia during the time of the study were invited to fill out the online surveys. On-line consents were obtained from the participants. Participants were allowed to terminate the survey at any time they wished. Participants who were undertraining, observership, volunteering, or were outside the kingdom were excluded from the study. Since the primary outcome of the study was to examine the levels of anxiety and depression among healthcare workers during and after lifting of the lockdown in Saudi Arabia, the survey was distributed at two time points. The first survey was circulated in May 2020. During this period, the total confirmed cases of COVID-19 exceeded 86,000 in Saudi Arabia; therefore, in order to tackle the rapid rise of cases, a nationwide lockdown (between 11 and 24-h) was implemented. The second survey was circulated in August 2020, 8 weeks after lifting of the lockdown restrictions and returning to “normalcy” in all cities around the kingdom (Supplementary Figure 1).

Surveys were distributed using social media platforms, internal e-mails, and/or e-mails by the Saudi Commission for Health Specialties (SCFHS) to a randomly selected group of registered practitioners together with a snowball recruiting technique. This allowed us to obtain an adequate sample from different regions in a wide variety of health specialties. The self-administered surveys took 10 min to complete and were available in Arabic and English languages.

### Measurements

The surveys included basic sociodemographic information including the participant's age, gender, marital status, income, nationality, education level and work status. Information regarding their direct exposure to COVID-19 patients, working in a COVID-19 designated site, being COVID-19 positive themselves or having a family member who is/was diagnosed with COVID-19 were also collected. Participants were also asked whether they were diagnosed for psychiatric disorders and/or chronic diseases in addition to their perception regarding their physical and mental health status compared to the time before the outbreak.

Anxiety and depressive symptoms were assessed via Generalized Anxiety Disorder (GAD-7) and Patient Health Questionnaire-9 (PHQ-9) scales; respectively. GAD-7 is a seven-item questionnaire and is most frequently used to measure, diagnose, and assess the severity of generalized anxiety disorder (GAD). The total scores—ranged from 0 to 21 - are interpreted as follows: no or minimal (0–4), mild (5–9), moderate (10–14), and severe (15–21) anxiety (14). PHQ-9 is a nine-item scale that is most commonly used for screening and diagnosing depression. PHQ-9 total scores—range from 0 to 27 - are interpreted as follows: normal (0–4), mild (5–9), moderate (10–14), moderately severe (15–19), and severe (20–27) depression (15). The threshold score of 10 for both scales has been shown to be a reasonable cutoff point for identifying cases of GAD and major depression (16, 17). Participants who had scores greater than the cutoff threshold were characterized as having severe symptoms.

### Statistical Analysis

Data analysis was performed using R Studio software; R Core Team (2019), R: A language and environment for statistical computing. R Foundation for Statistical Computing, Vienna, Austria. URL http://www.R-project.org/. We used the mean, standard deviation and range to describe numeric variables, proportions and odds ratio to describe categorical variables. We also used student *t*-test, ANOVA and chi-Square interferential statistical analysis. Furthermore, we constructed multiple linear regression analysis models to predict the change in anxiety and depression scale through the different stages of lockdown, using GAD-7 and PHQ-9 scores as the dependent variables for each model separately, and phases of lock down as the independent variables. The 95% confidence interval was determined, and the *p*-values were interpreted according to the American Statistical Association guidelines (18).

## Results

### Demographic Characteristics

A total of 947 healthcare workers who responded to two independent surveys were included in the analysis. The majority of the participants were females (53.3%), aged between 20 and 70 years (37 ± 8.9), living in Jeddah (37%), followed by Riyadh (20%) (Supplementary Figure 2), earning between 10,000 and 29,999 SAR/month (61.5%), married (66.5%), and had a tertiary level of education (80.6%). Saudi nationals accounted for 78 % of the participants. Three hundred and seventy-eight of the participants were allied healthcare professionals (AHPs) (39.8%), 192 nurses (20.2%), 171 physicians (18%), 113 medical trainees (12%), 46 dentists (4.8%), and 47 pharmacists (5%). Among these participants, 246 (25.9%) reported working in high-risk areas (i.e., working in emergency rooms, intensive care units, and/or isolation wards). A total of 359 participants (40%) reported to have direct contact with COVID-19 cases during the study, but only 49 participants (5%) were diagnosed with COVID-19. About one fourth of the participants (*n* = 250) reported living with elderly. Sixty-nine participants (7%) reported a current or a pre-existing psychiatric diagnosis, including anxiety and depression, and 21.6% reported to have a chronic disease.

The demographic characteristics of the responders of each survey are shown in Table 1. Out of the 947 healthcare workers who participated in the study, 553 had responded to the first survey (during the lockdown) and 392 had responded to the second survey (after lifting the lockdown). The majority of the responders to the first and the second surveys were females (53.5% and 53.6%; respectively, *p* = 0.9), did not report a current or a pre-existing psychiatric diagnosis (93 % and 92.2%, *p* = 0.64) or chronic diseases (59.2% and 40.8%, *p* = 0.96). However, non-Saudis composed 38% of the responders to the second survey and only 9.6% in the first survey (*p* < 0.001). The marital status of the responders to the first and the second surveys was also different where 33% of the first-survey responders were married and 62.9% were single compared to 23.5 and 71.9%; respectively, among the second-survey responders (*p* = 0.006). Further, rate of positive COVID-19 infection was higher among the second-survey responders (8.41%).

**Table 1:** Demographic and clinical characteristics of the participants.

	**Total sample (*n* = 947)**	**During lockdown (*n* = 553)**	**After lockdown (*n* = 392)**	***P*** **-value and statistics**
**Gender N (%)**				
Male	439 (46.4%)	257 (46.5%)	180 (45.9%)	χ^2^ = 0.009, df = 1, *p* = 0.9
Female	506 (53.3%)	296 (53.5%)	210 (53.6%)	
**Age** (Mean ± SD)	36.87 ± 8.87	34.78 ± 7.6	39.9 ± 9.72	*F*_1,941_ = 0.81, ***p<*** **0.001**
**Nationality**				
Saudis	740 (78%)	497 (90.4%)	243 (62.0%)	χ^2^ = 109.34, df = 1, ***p<*** **0.001**
Non-Saudis	202 (21.3%)	53 (9.6%)	149 (38.0%)	
**Monthly income**				
<10,000 SAR	227 (24.2%)	99 (18.1%)	128 (32.7%)	
10,000–29,999 SAR	584 (62.3%)	368 (67.4%)	216 (55.1%)	χ^2^ = 26.25, df = 2, ***p<*** **0.001**
30,000 SAR or more	127 (13.5%)	79 (14.5%)	48 (12.2%)	
**Marital status**				
Married	631 (66.5%)	183 (33.0%)	92 (23.5%)	
Single	275 (29%)	349 (62.9%)	282 (71.9%)	χ^2^ = 10.05, df = 2, ***p*** **=** **0.006**
Divorced**/**separated	39 (4.1%)	22 (4.0%)	17 (4.3%)	
**Education level**				
Diploma	173 (18.2%)	124 (22.5%)	49 (12.7%)	
Bachelor	425 (44.8%)	262 (47.6%)	163 (42.1%)	χ^2^ = 53.9, df = 3, ***p<*** **0.001**
Post-graduate degrees (MSc, PhD)	161 (17%)	55 (10%)	106 (27.4%)	
Advanced clinical training	178 (18.8%)	109 (19.8%)	69 (17.8%)	
**History of/current psychiatric diagnosis**				
Yes	69 (7%)	39 (7.0%)	30 (7.8%)	χ^2^ = 0.216, df = 1, *p* = 0.64
No	869 (91.6%)	516 (93.0%)	353 (92.2%)	
**Chronic disease**				
Yes	205 (21.6%)	121(21.8%)	84 (21.9%)	χ^2^ = 0.002, df = 1, *p* = 0.96
No	733 (77.2%)	434 (59.2%)	299 (40.8%)	
**Living with elderly**				
Yes	250 (26.3%)	167 (30.1%)	83 (21.2%)	χ^2^ = 0.002, df = 1, *p* = 0.96
No	697 (73.4%)	388 (69.9%)	309 (78.8%)	
**Working in COVID-19 designated site**				
Yes	452 (46.6%)	272 (49.0%)	180 (45.9%)	χ^2^ = 9.40, df = 1, ***p*** **=** **0.002**
No	495 (52.2%)	283 (51.0%)	212 (54.1%)	
**Infected with COVID-19**				
Yes	49 (5.2%)	16 (2.89%)	33 (8.41%)	χ^2^ = 14.34, df = 1, ***p<*** **0.001**
No	898 (94.6%)	539 (97.46%)	359 (91.5%)	

*Bold values denote statistical significance at p < 0.05*.

### Prevalence of Anxiety and Depression

The prevalence of severe anxiety, defined by GAD-7 total score of ≥ 10, among the entire study participants was 23.3%. The proportions of mild, moderate, and severe anxiety during the lockdown were 49.7, 40.9, and 9.4%; respectively, compared to the proportion after lifting of the lockdown 62.5, 21.2, and 16.3% (χ^2^ = 43.2, df = 1, *p* < 0.001). Sever anxiety (i.e., with threshold score of 10 or more) was more prevalent during the lockdown compared to the period when lockdown was lifted (25.9 *vs*. 19.6%, *p* = 0.02).

The prevalence of severe depression, defined by PHQ-9 total score of ≥ 10, was 27%. The proportions of minimal, mild, moderate, moderately severe, and severe depression were 38, 33.3, 13.9, 9.2, and 5.6% respectively, compared to the proportion after lifting of the lockdown 51.3, 24.8, 12.4, 5.6, and 5.9% (χ^2^ = 16.4, df = 4, *p* = 0.002). There was no difference in the distribution of severe depression (i.e., with threshold score of 10 or more) during and after lockdown (28.6 *vs*. 23.8%, *p* = 0.13).

### Association Between Demographic Characteristics and Levels of Anxiety & Depression

The average GAD-7 score across the participants during the two phases of the study was (6 ± 5.3); yet it was significantly higher during the lockdown (6.7 ± 5.4 *vs*. 5.1 ± 5.3, *p* < 0.001, 95% CI; 0.8, 2.2). These scores tended to be influenced by the participants' demographic characteristics. Table 2 summarizes the change in GAD-7 score during and after lockdown according to participants' characteristics. Women, participants with previous/current diagnosis of psychiatric disorders, working in COVID-19 designated sites, being in contact with COVID-19 patients, and those who were instructed to quarantine/isolate themselves demonstrated higher scores of GAD-7 during (*p* = 0.003, *p* = < 0.001, *p* = 0.005, *p* < 0.001, and *p* = 0.001) and after lockdown (*p* = 0.04, *p* < 0.001, *p* = 0.014, *p* = 0.04 and *p* < 0.001) respectively. Those who suffer from chronic diseases, working in high-risk areas (i.e., ER, ICU, and/or isolation wards), and those who were involved in the active screening process showed high levels of anxiety during the lockdown period (*p* = 0.001, *p* < 0.001, *p* < 0.001, respectively); but not after lifting of the lockdown (all *p* > 0.05). On the other hand, Saudi nationals and those living with elderly were more anxious after lifting of the lockdown (*p* < 0.001). Surprisingly, the level of anxiety did not differ significantly between those who were infected with COVID-19 and those who were not in both phases (*p* > 0.05); nevertheless, it was higher among the ones who reported having an infected family member during and after the lockdown (*p* = 0.004, *p* = 0.057 respectively). The impact on the anxiety level has also shown to vary based on the participants' occupation during and after the lockdown (*F*_5,549_ = 3.4, *p* = 0.004; *F*_5,386_ = 3.58, *p* = 0.004, respectively). *Post-hoc* comparisons using Tukey HSD test indicated that during the lockdown, the mean GAD-7 score for nurses (8.22 ± 6.33) was significantly different than the mean score of physicians and AHP (*p* = 0.005, *p* = 0.018). Although the level of anxiety among nurses was reduced after lifting of the lockdown, *post-hoc* comparisons indicated a trend toward significant differences between nurses and physicians (*p* = 0.056) (Table 2). A significant difference for the GAD-7 mean score between medical trainees and physicians was observed after the lockdown (*p* = 0.003). In addition, healthcare workers who perceived their physical and mental health status as being “worse/much worse” in comparison to the time before the outbreak showed a higher level of anxiety compared to the ones who thought that their health status did not change or became better (Table 2).

**Table 2:** Summary of the overall mean GAD-7 scores according to characteristics of the participants during and after lifting of the lockdown.

	**Mean GAD scores**					**Mean GAD scores for severe anxiety (**≥ **10)**				
	**During lockdown**		**After lockdown**			**During lockdown**		**After lockdown**		
	***N***	**Mean (SD)**	***N***	**Mean (SD)**	***p-*** **value^b^**	***N***	**Mean (SD)**	***N***	**Mean (SD)**	***p-*** **value^**b**^**
**Gender**										
Male	257	5.9 (4.86)	180	4.58 (5.35)	**0.007**	58	13.3 (2.9)	28	15.1 (3.6)	**0.012**
Female	296	7.2 (5.68)	210	5.69 (5.28)	**0.002**	85	14.8 (3.5)	49	13.6 (3.3)	**0.043**
*p-*value^a^		**0.003**		**0.04**			**0.004**		**0.06**	
**History of current psychiatric diagnosis**										
Yes	39	9.9 (6.17)	30	9.4 (5.34)	0.69	22	14.5 (3.39)	15	13.7 (3.6)	0.52
No	516	6.4 (5.2)	353	4.79 (5.17)	** <0.001**	122	14.2 (3.4)	59	14.3 (3.4)	0.74
*p-*value^a^		** <0.001**		** <0.001**			0.70		0.5	
**Chronic disease**										
Yes	121	8.07 (5.85)	84	5.9 (5.37)	**0.008**	45	14.6 (3.3)	22	13.5(2.6)	0.18
No	434	6.26 (5.15)	299	4.9 (5.3)	** <0.001**	99	14.08 (3.40)	52	14.5 (3.7)	0.48
*p-*value^a^		**0.001**		**0.10**			0.39		0.29	
**Working in COVID-19 designated site**										
Yes	272	7.31 (5.45)	180	5.89 (5.71)	**0.008**	81	14.3 (3.3)	43	14.5 (3.5)	0.75
No	283	6.0 (5.2)	212	4.6 (4.91)	**0.002**	63	14.1 (3.4)	34	13.7 (3.3)	0.59
*p-*value^a^		**0.005**		**0.014**			0.7		0.32	
**Living with elderly**										
Yes	167	6.9 (5.5)	83	7.25 (6.24)	0.73	52	13.8 (3.3)	25	15.5 (3.5)	**0.045**
No	388	6.52 (5.3)	309	4.6 (4.9)	** <0.001**	92	14.4 (13.39)	52	13.5 (3.3)	0.11
*p-*value^a^		0.35		** <0.001**			0.26		**0.018**	
**Infected with COVID-19**										
Yes	16	7.25 (5.59)	33	4.9 (4.9)	0.153	5	3.6 (4.5)	9	12 (2.1)	0.48
No	539	6.64 (5.36)	359	5.1 (5.37)	** <0.001**	139	14.2 (3.3)	68	14.4 (3.5)	0.68
*p-*value^a^		0.65		0.81			0.66		0.04	
**In contact with COVID-19 patients**										
Yes	187	8.3 (5.77)	172	5.8 (5.4)	** <0.001**	70	14.5 (3.5)	38	14.3 (3.6)	0.83
No	368	5.8 (4.94)	220	4.6 (5.18)	**0.008**	74	13.9 (3.2)	39	13.9 (3.3)	0.98
*p-*value^a^		** <0.001**		**0.04**			0.30		0.5	
**Nationality**										
Saudi	497	6.63 (5.29)	243	6.03 (5.58)	0.161	130	14.08 (3.29)	59	14.4 (3.4)	0.45
Non-Saudi	53	7.13 (6.14)	149	3.77 (4.58)	** <0.001**	14	15.7 (4.02)	18	13.2 (3.4)	**0.06**
*p-*value^a^		0.51		** <0.001**			0.08		0.18	
**Occupation**										
Physician	94	5.57 (5.2)	77	3.38 (4.3)	**0.003**	17	14.7 (3.1)	5	15.6 (4.8)	0.65
Nurse	113	8.22 (6.33)	79	5.7 (5.3)	**0.004**	43	15.1 (3.6)	19	14 (2.8)	0.21
Medical trainee	71	7.32 (5.15)	42	7.14 (5.6)	0.86	24	13.2 (3.4)	14	13.7 (3.7)	0.65
Dentist	20	5.45 (5.56)	26	6.19 (6.3)	0.68	3	17 (3.6)	6	16.17 (2.6)	0.70
Pharmacist	27	6.26 (4.9)	20	6.20 (6.01)	0.9	5	14.6 (2.8)	5	15 (3)	0.83
Allied health care professional (AHP)	230	6.27 (4.8)	148	4.93 (5.16)	**0.011**	52	13.5 (3.06)	28	13.6 (3.7)	0.87
*p-*value^a^		**0.004**		**0.004**			0.07		0.5	
**Working in high-risk area**										
Yes	161	8.18 (5.78)	85	5.25 (5.47)	** <0.001**	59	14.5 (3.5)	17	14.2 (4.05)	0.79
No	394	6.03 (5.05)	307	5.15 (5.3)	**0.026**	85	14.02 (3.28)	60	14.15 (3.3)	0.82
*p-*value^a^		** <0.001**		0.88			0.35		0.8	
**Confirmed cases among family members**										
Yes	22	9.86 (6.48)	102	6.04 (5.64)	**0.006**	11	15.45 (2.8)	26	14.3 (3.8)	0.38
No	533	6.52 (5.27)	290	4.8 (5.19)	** <0.001**	133	14.14 (3.4)	51	14.1 (3.3)	0.96
*p-*value^a^		**0.004**		**0.057**			0.2		0.8	
**Experienced quarantine/isolation**										
Yes	107	8.2 (5.9)	107	6.7 (5.7)	** <0.001**	37	15.1 (3.6)	33	14.1 (3.4)	0.25
No	448	6.29 (5.16)	285	4.5 (5.04)	** <0.001**	107	13.9 (3.2)	44	14.2 (3.5)	0.64
*p-*value^a^		**0.001**		** <0.001**			0.07		0.8	
**Active screener**										
Yes	51	9.18 (6.25)	52	4.3 (4.8)	** <0.001**	20	15.9 (3.5)	7	13.8 (3.6)	0.20
No	500	6.41 (5.19)	340	5.3(5.3)	**0.003**	123	13.9 (3.3)	70	14.2 (3.4)	0.62
*p-*value^a^		** <0.001**		**0.22**			0.018		0.7	
**Perceived physical health**										
Almost not changed	305	5.2 (4.68)	172	3.5 (4.07)	** <0.001**	45	14.1 (3.6)	17	12.7 (2.8)	0.11
Better	92	6.58 (5.65)	52	3.38 (4.4)	** <0.001**	25	14.3 (3.7)	5	14.2 (3.6)	0.9
Worse	133	8.84 (5.2)	67	8.21 (5.3)	0.421	55	14.09 (3.03)	26	13.9 (3.1)	0.86
Much Worse	25	12.7 (4.97)	15	13.27 (5.2)	0.743	19	14.7 (3.4)	12	15.08 (3.9)	0.79
*p-*value^a^		** <0.001**		** <0.001**			0.9		0.3	
**Perceived mental health**										
Almost not changed	222	4.04 (4.1)	142	2.6 (3.3)	**0.001**	18	14.6 (3.7)	8	12.2 (2.7)	0.11
better	88	6.26 (5.66)	54	3.02 (4.38)	** <0.001**	23	14.1 (3.9)	4	15.2 (4.5)	0.62
Worse	187	8.07 (4.65)	86	7.83 (4.64)	0.681	58	13.8 (3.1)	29	13.34 (2.8)	0.49
Much worse	58	12.7 (4.85)	24	13.25 (4.8)	0.647	45	14.6 (3.2)	19	15.05 (3.6)	0.66
*p-*value^a^		** <0.001**		** <0.001**			0.6		0.11	

*Bold values denote statistical significance at p < 0.05*.

*p-value^a^ describes the statistical difference between groups within the same lockdown period (e.g., males vs. females)*.

*p-value^b^ describes the statistical difference within the same group but at different time points (e.g., males before vs. after lockdown)*.

In terms of the scores during and after the lockdown for each group, an overall significant reduction was observed. For example, the total GAD-7 scores were significantly reduced for both men and women during and after the lockdown (*p* = 0.007, *p* = 0.002). A similar pattern of a consistent drop in GAD-7 score after lifting of lockdown was observed in all categories except in participants with a pre-existing/current psychiatric disorder, those who were living with elderly, and those who were infected with COVID-19 (all *p* > 0.05). In addition, medical trainees, dentists, and pharmacists did not show a significant change in GAD-7 scores during the two phases. It was also observed that the mean anxiety score was reduced among non-Saudis after lifting of the lockdown.

Similar analyses were performed on PHQ-9 scale (Table 3). The average PHQ-9 score across the participants during the two phases of the study was 6.9 ± 6.24; yet it was significantly higher during the lockdown (7.4 ± 6.11 *vs*. 6.1 ± 6.4, *p* = 0.002). Similarly, women participants with mental illnesses, and in contact with COVID-19 patients, who perceived their physical and mental health as “worse/much worse” were likely to demonstrate significantly higher scores on PhQ-9 during (all *p* < 0.001) and after lockdown (*p* = 0.02, *p* < 0.001, *p* = 0.002, *p* < 0.001) respectively. Also, PHQ-9 score was shown to differ based on the participants' occupation during and after the lockdown (*F*_5,549_ = 3.7, *p* = 0.002; *F*_5,300_ = 6.5, *p* < 0.001, respectively), where nurses and medical trainees expressed the highest scores. *Post-hoc* comparisons indicated that, during the lockdown, the mean PHQ-9 score for nurses was significantly different than AHP (*p* = 0.034) and marginally different than physicians (*p* = 0.056). Significant differences were also observed between medical trainees and physicians (*p* = 0.043) and AHP (*p* = 0.032). Level of depression was reduced among nurses after the lockdown but not for medical trainees.

**Table 3:** Summary of the overall mean PHQ-9 scores according to characteristics of the participants during and after lifting of the lockdown.

	**Mean PHQ-9 scores**					**Mean PHQ-9 scores for severe anxiety (**≥ **10)**				
	**During lockdown**		**After lockdown**			**During lockdown**		**After lockdown**		
	***N***	**Mean (SD)**	***N***	**Mean (SD)**	***p-*** **value** ^b^	***N***	**Mean (SD)**	***N***	**Mean (SD)**	***p-*** **value** ^b^
**Gender**										
Male	257	6.3 (5.8)	146	5.2 (6.2)	0.09	64	14.8 (4.2)	26	16.4 (5.4)	0.14
Female	296	8.5 (6.07)	159	6.9 (6.4)	**0.011**	94	15.8 (4.4)	47	14.9 (4.8)	0.30
*p-*value^a^		**<** **0.001**		**0.02**			0.12		0.22	
**History of current psychiatric diagnosis**										
Yes	39	13.2 (7.6)	25	13.16 (8.4)	0.96	24	18.3 (4.6)	16	18 (6.1)	0.85
No	516	7.03 (5.7)	247	5.4 (5.7)	** <0.001**	135	14.9 (4.1)	56	14.6 (4.4)	0.71
*p-*value^a^		** <0.001**		** <0.001**			** <0.001**		**0.018**	
**Chronic disease**										
Yes	121	8.2 (6.3)	64	6.7 (5.8)	0.09	41	15.5 (4.7)	20	14.1 (3.5)	0.17
No	434	7.2 (6.0)	235	5.9 (6.4)	**0.008**	118	15.3 (4.3)	52	15.8 (5.4)	0.54
*p-*value^a^		0.10		0.40			**0.7**		**0.18**	
**Working in COVID-19 designated site**										
Yes	272	8.03 (6.29)	137	6.7 (6.9)	0.06	87	15.6 (4.4)	39	15.8 (5.5)	0.88
No	283	6.93 (5.9)	169	5.5 (5.9)	**0.014**	72	15.10 (4.4)	34	15.09 (4.4)	0.9
*p-*value^a^		**0.034**		0.09			0.4		0.54	
**Living with elderly**										
Yes	167	8.4 (6.3)	67	7.9 (7.7)	0.57	57	15.7 (4.5)	21	17.8 (5.6)	0.09
No	388	7.0 (5.9)	239	5.5 (5.8)	**0.002**	102	15.2 (4.3)	52	14.5 (4.5)	0.35
*p-*value^a^		**0.010**		**0.006**			0.4		**0.012**	
**Infected with COVID-19**										
Yes	16	6.13 (5.4)	24	6.1 (7.1)	1.00	3	14 (6.08)	6	16.17 (7.02)	0.65
No	539	7.5 (6.13)	282	6.06 (6.3)	**0.002**	156	15.4 (4.4)	67	15.4 (4.9)	0.97
*p-*value^a^		0.37		0.9			0.5		0.73	
**In contact with COVID-19 patients**										
Yes	187	9.2 (6.8)	129	7.4 (7.05)	**0.025**	75	16.2 (4.5)	39	16.2 (5.5)	0.9
No	368	6.5 (5.4)	177	5.09 (5.6)	**0.003**	84	14.6 (4.2)	34	14.5 (4.3)	0.9
*p-*value^a^		** <0.001**		**0.002**			**0.018**		0.16	
**Nationality**										
Saudi	497	7.4 (6.0)	192	7.0 (6.7)	0.39	147	15.14 (4.2)	57	15.6 (5.2)	0.48
Non-Saudi	53	7.5 (7.1)	114	4.4 (5.2)	**0.006**	12	18.8 (5.1)	16	14.7 (4.3)	**0.03**
*p-*value^a^		0.9		**0.001**			**0.005**		0.5	
**Occupation**										
Physician	94	6.5 (6.2)	62	4.08 (4.34)	**0.005**	24	15.4 (4.6)	6	13.8 (3.6)	0.44
Nurse	113	8.8 (6.5)	63	6.8 (7.02)	**0.06**	37	16.7 (4.07)	17	16.2 (5.5)	0.72
Medical trainee	71	9.2 (6.3)	36	10.9 (8.01)	0.24	30	15.47 (4.3)	20	16.4 (6.4)	0.57
Dentist	20	6.4 (5.1)	20	5.10 (5.5)	0.42	4	14.7 (3.4)	4	14.5 (2.08)	0.90
Pharmacist	27	6.6 (5.6)	14	6.07 (6.3)	0.77	6	15.6 (2.5)	3	15.6 (6.02)	1.00
Allied health care professional (AHP)	230	6.8 (5.7)	111	5.3 (5.7)	**0.025**	58	14.5 (4.7)	23	14.7 (4.07)	0.88
*p-*value^a^		**0.002**		** <0.001**			0.31		0.8	
**Working in high-risk area**										
Yes	161	9.2 (6.6)	64	6.8 (7.1)	**0.016**	65	16.06 (4.4)	21	15.2 (5.7)	0.57
No	394	6.7 (5.7)	242	5.8 (6.1)	0.07	94	14.9 (4.3)	52	15.5 (4.8)	0.45
*p-*value^a^		** <0.001**		0.30			0.126		0.8	
**Confirmed cases among family members**										
Yes	22	10 (6.8)	76	7.2 (6.4)	0.08	10	15.6 (5.6)	27	14.2 (4.7)	0.47
No	533	7.3 (6.07)	230	5.6 (6.3)	** <0.001**	149	15.4 (4.3)	46	16.2 (5.1)	0.30
*p-*value^a^		**0.04**		0.07			0.8		0.11	
**Experienced quarantine/isolation**										
Yes	107	8.4 (6.3)	83	7.9 (7.1)	0.61	38	15.5 (4.3)	31	15.5 (5.6)	0.88
No	448	7.2 (6.04)	223	5.3 (5.9)	** <0.001**	121	15.3 (4.4)	42	15.4 (4.7)	0.92
*p-*value^a^		0.06		**0.001**			0.8		0.9	
**Active screening**										
Yes	51	9.2 (7.5)	42	5.8 (6.7)	**0.026**	20	17.3 (4.8)	9	16.3 (6.2)	0.65
No	500	7.3 (5.9)	264	6.1 (6.3)	**0.009**	139	15.1 (4.3)	64	15.3 (4.9)	0.75
*p-*value^a^		**0.029**		0.8			**0.04**		0.5	
**Perceived physical health**										
Almost not changed	305	5.7 (5.1)	172	4.2 (4.5)	**0.002**	52	14.7 (4.2)	21	13.2 (4.1)	0.19
Better	92	6.6 (6.1)	52	3.4 (5.1)	**0.002**	26	14.6 (4.6)	5	16.4 (4.3)	0.43
Worse	133	10.4 (5.8)	67	10.10 (6.2)	0.67	59	15.7 (4.4)	34	15.1 (4.2)	0.66
Much worse	25	15.8 (5.2)	15	7.3 (18.07)	0.31	22	17.05 (4.3)	13	19.6 (6.5)	0.22
*p-*value^a^		** <0.001**		** <0.001**			0.13		**0.003**	
**Perceived mental health**										
Almost not changed	22	4.6 (4.6)	142	3.5 (3.9)	**0.019**	26	14.3 (4.6)	15	11.9 (2.8)	**0.048**
Better	88	6.4 (6.3)	54	3.1 (4.1)	** <0.001**	20	15.9 (4.7)	2	16.5 (7.7)	0.88
Worse	187	8.8 (5.0)	86	9.09 (6.38)	0.78	65	14.4 (4.0)	36	15.2 (4.4)	0.33
Much worse	58	15.12 (5.9)	24	16.63 (6.69)	0.31	48	17.1 (4.2)	20	18.4 (5.7)	0.36
*p-*value^a^		** <0.001**		** <0.001**			**0.006**		**0.002**	

*Bold values denote statistical significance at p < 0.05*.

*p-value^a^ describes the statistical difference between groups within the same lockdown period (e.g., males vs. females)*.

*p-value^b^ describes the statistical difference within the same group but at different time points (e.g., males during vs. after lockdown)*.

On the contrary for GAD-7 findings, working in COVID-19 designated sites, in high-risk areas, being an active screener and having an infected family member (*p* = 0.034, *p* < 0.001, *p* = 0.029, *p* = 0.04) were associated with higher total PHQ-9 scores but only during the lockdown. Saudis and participants who were asked to isolate themselves showed higher level of depression after lifting of lockdown (*p* < 0.001). In terms of changes in the scores within the groups during and after the lockdown, an overall significant reduction was observed except for some groups. Similar to GAD-7 scale, the mean score for PHQ-9 also did not change significantly after lifting of the lockdown among medical trainees, dentists, pharmacists, participants with a psychiatric diagnosis or chronic diseases, the ones who were infected with COVID-19, or who were not working in high-risk areas (all *p* > 0.05) (Table 3). Interestingly, these findings were not observed among the surveys' respondents when the analysis was restricted to participants with severe anxiety and depression.

### Predictors of Anxiety and Depression During and After Lockdown

A linear regression analysis was conducted to assess whether the sociodemographic characteristics and the variables were related to the COVID-19 outbreak and could be significant predictors for the psychological outcomes (the GAD-7 and PHQ-9 scores). The “Enter” variable selection method was chosen for the linear regression model, which included all the selected predictors. Assumptions of homoscedasticity and multicollinearity were met and all predictors in the regression model presented a tolerance of more than 0.1 and VIFs < 10. The results of the linear regression model, for GAD-7, were significant (*F*_25,921_ = 21.3, *p* < 0.001, *R*^2^ = 0.37, adjusted *R*^2^ = 0.35), indicating that approximately 37% of the variance in the level of anxiety could be explained by the studied factors. Lockdown status, gender, occupation, psychiatric diagnosis, chronic diseases, experiencing quarantine/self-isolation, the perceived physical and mental health status were found to be the factors to influence anxiety among healthcare workers. A significant drop in GAD-7 scores (1.2 points less, *p* < 0.001, 95 % CI; 0.54, 1.92) was noticed when the lockdown was lifted. Females, participants with psychiatric diagnosis and chronic diseases, nurses, participants who experienced quarantine or self-isolation, and who perceived their physical and mental health as much worse compared to the time before the pandemic showed higher level of psychological anxiety (Table 4A).

**Table 4A:** A. Regression coefficients for Generalized Anxiety Disorder-7 (GAD-7) as an outcome with socio-demographic and COVID-19 related variables as predictors.

	**GAD-7** **(***R***^**2**^ = 0.37)**					**GAD-7—During Lockdown** **(***R***^**2**^ = 0.36)**					**GAD-7—After Lockdown** **(***R***^**2**^ = 0.41)**				
	**β**	**SEM**	***t*** **value**	***p*** **-value**	**95% CI**	**β**	**SEM**	***t*** **value**	***p*** **-value**	**95% CI**	**β**	**SEM**	***t*** **value**	***p*** **-value**	**95% CI**
**Lockdown status**															
During lockdown	1.234	0.351	3.517	** <0.001**	0.54, 1.92										
After lockdown	Reference														
**Gender**															
Male	−0.81	0.299	−2.738	**0.006**	−1.40, −0.23	−0.879	0.394	−2.232	**0.026**	−1.65, −0.1	−0.693	0.468	−1.480	0.140	−1.61, 0.22
Female	Reference					Reference					Reference				
**History of current psychiatric diagnosis**															
Yes	2.585	0.564	4.586	** <0.001**	1.47, 3.69	2.83	0.753	3.763	** <0.001**	1.35, 4.31	1.627	0.889	1.831	0.068	−0.12, 3.37
No	Reference					Reference					Reference				
**Chronic disease**															
Yes	1.050	0.350	3.004	**0.003**	0.36,1.73	1.395	0.457	3.055	**0.002**	0.49, 2.29	0.425	0.545	0.780	0.436	−0.64, 1.49
No	Reference					Reference					Reference				
**Working in COVID-19 designated site**															
Yes	0.292	0.328	0.891	0.373	−0.35, 0.93	−0.19	0.437	−0.445	0.657	−1.05, 0.66	0.990	0.502	1.972	**0.049**	0.003, 1.97
No	Reference					Reference					Reference				
**Living with elderly**															
Yes	0.476	0.339	1.404	0.161	−0.18, 1.14	0.082	0.424	0.193	0.847	−0.75, 0.91	1.313	0.577	2.274	**0.024**	0.17, 2.44
No	Reference					Reference					Reference				
**Infected with COVID-19**															
Yes	−1.25	0.726	−1.724	0.085	−2.67, 0.17	0.168	1.192	0.141	0.888	−2.17, 2.5	−1.859	0.930	−1.999	**0.046**	−3.68, −0.03
No	Reference					Reference					Reference				
**In contact with COVID-19 patients**															
Yes	0.584	0.356	1.641	0.101	−0.11, 1.28	0.861	0.484	1.779	0.076	−0.09, 1.81	0.065	0.538	0.122	0.903	−0.99, 1.12
No	Reference					Reference					Reference				
**Nationality**															
Saudi	0.652	0.414	1.575	0.115	−0.16, 1.46	0.527	0.691	0.762	0.446	−0.83, 1.88	0.841	0.519	1.621	0.106	−0.17, 1.86
Non–Saudi	Reference					Reference					Reference				
**Occupation**															
Physician	−1.22	0.419	−2.925	**0.004**	−2.04, −0.40	−1.28	0.550	−2.336	**0.020**	−2.36, −0.2	−0.909	0.647	−1.404	0.161	−2.18, 0.36
Nurse	0.855	0.421	2.031	**0.043**	0.02, 1.68	0.685	0.550	1.247	0.213	−0.39, 1.76	0.940	0.655	1.435	0.152	−0.34, 2.22
Medical trainee	−0.60	0.487	−1.237	0.216	−1.56, 0.35	−0.95	0.619	−1.543	0.123	−2.17, 0.26	−0.398	0.801	−0.497	0.620	−1.97, 1.17
Dentist	0.495	0.691	0.716	0.474	−0.86, 1.85	−1.22	1.038	−1.176	0.240	−3.25, 0.81	1.750	0.927	1.887	0.060	−0.07, 3.57
Pharmacist	0.969	0.679	1.426	0.154	−0.36, 2.30	0.474	0.892	0.531	0.596	−1.27, 2.22	1.455	1.043	1.394	0.164	−0.59, 3.50
AHP	–	–	–	–	–	–	–	–	–	–	–	–	–	–	–
**Working in high-risk area**															
Yes	0.589	0.370	1.593	0.112	−0.13, 1.31	1.08	0.472	2.289	**0.022**	0.15, 2.0	0.174	0.603	0.289	0.773	−1.01, 1.36
No	Reference					Reference					Reference				
**Family member infected with COVID-19**															
Yes	0.764	0.472	1.618	0.106	−0.16, 1.69	2.15	0.976	2.209	**0.028**	0.23, 4.07	0.270	0.547	0.494	0.621	−0.80, 1.34
No	Reference					Reference					Reference				
**Experienced quarantine/isolation**															
Yes	1.040	0.397	2.623	**0.009**	0.26, 1.81	0.647	0.537	1.205	0.229	−0.40, 1.7	1.479	0.599	2.467	**0.014**	0.3, 2.65
No	Reference					Reference					Reference				
**Active screening**															
Yes	0.621	0.507	1.224	0.221	−0.37, 1.61	1.862	0.714	2.608	**0.009**	0.45, 3.26	−0.459	0.735	−0.625	0.532	−1.90, 0.98
No	Reference					Reference					Reference				
**Perceived physical health**															
Almost not changed	–	–	–	–	–	–	–	–	–	–	−2.911	0.599	−4.860	** <0.001**	−4.08, −1.73
Better	0.652	0.523	1.246	0.213	−0.37, 1.67	0.773	0.667	1.159	0.247	−0.53, 2.08	−2.520	0.983	−2.562	**0.011**	−4.45, −0.58
Worse	1.418	0.418	3.395	**0.001**	0.59, 2.23	1.20	0.520	2.306	**0.021**	0.17, 2.22	−0.841	0.855	−0.983	0.326	−2.52, 0.84
Much worse	2.577	0.847	3.041	**0.002**	0.91, 4.24	2.45	1.104	2.225	**0.027**	0.28, 4.62	0.668	1.465	0.456	0.649	−2.21, 3.54
**Perceived mental health**															
Almost not changed	−2.77	0.564	−4.916	** <0.001**	−3.87, −1.66	–	–	–	–	–	–	–	–	–	–
Better	−2.00	0.729	−2.751	**0.006**	−3.43, −0.57	1.23	0.705	1.749	0.081	−0.15, 2.62	−0.055	0.850	−0.065	0.948	−1.72, 1.61
Worse	0.742	0.624	1.190	0.235	−0.48, 1.96	3.37	0.489	6.904	** <0.001**	2.41, 4.33	3.675	0.659	5.575	** <0.001**	2.37, 4.97
Much worse	4.040	0.822	4.917	** <0.001**	2.42, 5.65	6.43	0.808	7.965	** <0.001**	4.85, 8.02	7.030	1.150	6.115	** <0.001**	4.76, 9.29

*Bold values denote statistical significance at p < 0.05*.

Interestingly, these predictors tend to vary based on the time of the pandemic. Gender, suffering from chronic diseases, working in high-risk areas, having an infected family member, being involved in active screening, and perceiving physical health as worse/much worse, have shown to be significant predictors for anxiety only during the time of the lockdown. On the other hand, working in COVID-19 designated sites, living with elderly, not infected with COVID-19, experiencing quarantine/isolation were significant predictors after lifting of the lockdown. Perceiving mental health status as worse/much worse was shown to be a predictor during both phases (Table 4A).

With respect to the depression scores, the results of the linear regression model were also significant (*F*_24,836_ = 28.7, *p* < 0.001, *R*^2^ = 0.45, adjusted *R*^2^ = 0.43), indicating that approximately 45% of the variance in the level of depression was explainable by the studied factors. Similar to GAD-7, a significant drop in PHQ-9 scores (0.94 points less, *p* = 0.014, 95 % CI; 0.19, 1.7) was detected when the lockdown was lifted. Further, females, participants with psychiatric diagnosis, living with elderly, being in contact with COVID-19 cases, working in high-risk areas, and the ones who perceived their physical and mental health as “worse/much worse” compared to the time before the pandemic showed higher level of psychological depression (Table 4B). Most of these predictors, i.e., gender, psychiatric diagnosis, being in contact with COVID-19 patients, and perceived physical and mental health tend to be significant regardless of the phase of the lockdown. Living with elderly and working in high-risk areas were significant predictors for depression but only during the lockdown (Table 4B).

**Table 4B:** B. Regression coefficients for Patient Health Questionnaire-9 (PHQ-9) as an outcome with socio-demographic and COVID-19 related variables as predictors.

	**PHQ-9** **(***R***^**2**^ = 0.45)**					**PHQ-9–during lockdown** **(***R***^**2**^ = 0.36)**					**PHQ-9–after lockdown** **(***R***^**2**^ = 0.41)**				
	**β**	**SEM**	***t*** **value**	***p*** **-value**	**95% CI**	**β**	**SEM**	***t*** **value**	***p*** **-value**	**95% CI**	**β**	**SEM**	***t*** **value**	***p*** **-value**	**95% CI**
**Lockdown status**															
During lockdown	0.948	0.383	2.473	**0.014**	0.19, 1.7										
After lockdown	Reference														
**Gender**															
Male	−1.514	0.339	−4.470	** <0.001**	−2.17, −0.84	−1.64	0.431	−3.813	** <0.001**	−2.49, −0.79	−1.34	0.581	−2.313	**0.021**	−2.48, −0.2
Female	Reference					Reference					Reference				
**History of current psychiatric diagnosis**															
Yes	4.555	0.634	7.186	** <0.001**	3.31, 5.79	5.103	0.825	6.182	** <0.001**	3.48, 6.72	3.031	1.094	2.772	**0.006**	0.87, 5.184
No	Reference					Reference					Reference				
**Chronic disease**															
Yes	0.044	0.396	0.110	0.912	−0.73, 0.82	0.341	0.500	0.683	0.495	−0.64, 1.32	−0.603	0.674	−0.894	0.372	−1.93, 0.72
No	Reference					Reference					Reference				
**Working in COVID-19 designated site**															
Yes	−0.442	0.372	−1.187	0.236	−1.17, 0.28	−0.540	0.479	−1.128	0.260	−1.48, 0.4	−0.270	0.613	−0.44	0.66	−1.47, 0.93
No	Reference					Reference					Reference				
**Living with elderly**															
Yes	0.767	0.379	2.025	**0.043**	0.02, 1.51	0.976	0.464	2.103	**0.036**	0.06, 1.88	0.413	0.693	0.596	0.552	−0.95, 1.77
No	Reference					Reference					Reference				
**Infected with COVID-19**															
Yes	−1.37	0.857	−1.605	0.109	−3.05, 0.30	−1.21	1.30	−0.929	0.353	−3.78, 1.35	−1.68	1.162	−1.453	0.147	−3.97, 0.59
No	Reference					Reference					Reference				
**In contact with COVID-19 patients**															
Yes	1.243	0.408	3.048	**0.002**	0.44, 2.04	1.050	0.530	1.979	**0.048**	0.008, 2.09	1.54	0.674	2.298	**0.022**	0.22, 2.87
No	Reference					Reference					Reference				
**Nationality**															
Saudi	0.524	0.478	1.095	0.274	−0.41, 1.46	0.268	0.757	0.354	0.723	−1.21, 1.75	0.733	0.624	1.176	0.241	−0.49, 1.96
Non-Saudi	Reference					Reference					Reference				
**Occupation**															
Physician	−0.930	0.474	−1.964	**0.050**	−1.85, 0	−0.874	0.603	−1.450	0.148	−2.05, 0.31	−0.823	0.794	−1.036	0.301	−2.38, 0.74
Nurse	0.850	0.474	1.793	0.073	−0.08, 1.78	0.710	0.602	1.179	0.239	−0.47, 1.89	0.891	0.792	1.125	0.261	−0.66, 2.44
Medical trainee	0.658	0.545	1.208	0.227	−0.41, 1.72	0.069	0.678	0.101	0.919	−1.26, 1.40	1.713	0.967	1.771	0.078	−0.19, 3.61
Dentist	−0.454	0.801	−0.567	0.571	−2.02, 1.11	−1.09	1.137	−0.966	0.335	−3.33, 1.13	0.348	1.151	0.302	0.763	−1.91, 2.61
Pharmacist	0.422	0.780	0.541	0.589	−1.10, 1.95	0.077	0.978	0.078	0.938	−1.84, 1.99	0.641	1.324	0.484	0.629	−1.96, 3.24
AHP	–	–	–	–	–	–	–	–	–	–	–	–	–	–	–
**Working in high-risk area**															
Yes	1.01	0.42	2.420	**0.016**	0.19, 1.84	1.34	0.517	2.604	**0.009**	0.33, 2.36	0.551	0.763	0.722	0.471	−0.95, 2.05
No	Reference					Reference					Reference				
**Family member infected with COVID-19**															
Yes	0.680	0.563	1.209	0.227	−0.42, 1.78	1.370	1.069	1.281	0.201	−0.73, 3.47	0.187	0.676	0.277	0.782	−1.14, 1.51
No	Reference					Reference								
**Experienced quarantine/isolation**															
Yes	0.13	0.44	0.298	0.766	−0.74, 1.01	−0.095	0.588	−0.161	0.872	−1.25, 1.06	0.700	0.724	0.967	0.334	−0.72, 2.12
No	Reference					Reference					Reference				
**Active screening**															
Yes	0.76	0.57	1.333	0.183	−0.36, 1.88	1.21	0.782	1.559	0.120	−0.31, 2.75	0.425	0.888	0.479	0.633	−1.32, 2.17
No	Reference					Reference					Reference				
**Perceived physical health**															
Almost not changed	–	–	–	–	–	–	–	–	–	–	–	–	–	–	–
Better	0.387	0.564	0.686	0.493	−0.72, 1.49	0.487	0.731	0.665	0.506	−0.95, 1.92	0.383	0.918	0.417	0.677	−1.42, 2.19
Worse	2.701	0.451	5.995	** <0.001**	1.81, 3.58	2.370	0.570	4.159	** <0.001**	1.25, 3.48	3.155	0.756	4.172	** <0.001**	1.66, 4.64
Much worse	5.278	0.913	5.778	** <0.001**	3.48, 7.07	5.006	1.21	4.138	** <0.001**	2.6, 7.38	6.105	1.480	4.126	** <0.001**	3.19, 9.01
**Perceived mental health**															
Almost not changed	–	–	–	–	–	–	–	–	–	–	–	–	–	–	
Better	0.307	0.584	0.525	0.600	−0.84, 1.45	0.884	0.773	1.144	0.253	−0.63, 2.40	−0.736	0.910	−0.809	0.419	−2.52, 1.05
Worse	3.201	0.422	7.583	** <0.001**	2.37, 4.03	3.041	0.536	5.679	** <0.001**	1.98, 4.09	3.576	0.707	5.059	** <0.001**	2.18, 4.96
Much worse	7.001	0.704	9.944	** <0.001**	5.61, 8.38	6.703	0.885	7.571	** <0.001**	4.96, 8.44	7.803	1.236	6.312	** <0.001**	5.37, 10.23

*Bold values denote statistical significance at p < 0.05*.

## Discussion

The current study sought to measure the magnitude of depression and anxiety during and after the lockdown restrictions among healthcare professionals across different healthcare settings in Saudi Arabia. It also examined whether some individuals could be more adversely affected by the COVID-19 pandemic than others by looking at the association between different demographic characteristics and the psychological impact.

We observed a prevalence of significant depression and anxiety (27 and 23% respectively) among healthcare professionals. This is in line with a recent systematic review (19) which reported prevalence rates of 12.1–55.89% for depression and 24.1–67.55% for anxiety. For the regional comparison, the reported prevalence rates of depression and anxiety among healthcare professionals in Saudi Arabia ranged between 55.2 and 69% for depression and 31.5–58.9% for anxiety (20–24). The different methods used in various studies can explain the higher prevalence rates observed compared to the current study. Explanation of these high rates also include not accounting for the period after easing of the lockdown restrictions and assessing depression and anxiety during the first few months of the pandemic.

Across both periods of the lockdown, higher levels of depression and anxiety were found in women, who were at a greater risk of infection due to working in COVID-19 designated sites or in contact with COVID-19 patients, or working in high-risk places (ER, ICU, and/or isolated wards), had a current or a previous history of psychiatric disorder, were living with elderly and the ones who were instructed to quarantine/isolate themselves. Similar to our results, systematic reviews (19, 25, 26) and a meta-analysis (27) revealed higher depression and anxiety levels among female healthcare professionals and ones working in the areas with high rates of COVID-19 cases.

We noticed that the depression and anxiety levels were higher in medical trainees and nurses compared to other specialties. Nurses have consistently shown higher rates of depression and anxiety in systematic reviews, although medical trainees were not specifically targeted in the systematic reviews (19, 25, 26). Given the challenges faced by medical trainees during the pandemic such as limited patient contacts, non-campus activities, online assessment and exams, and restricted clinical rotations, significant stress and burnout were reported (28–30). In fact, longer professional experience has been reported as potentially protective factor to develop psychopathological distress in healthcare workers (31).

We did not detect significant differences between those who were infected with the virus and those who were not. On the contrary, the ones with family members who had been diagnosed with COVID-19 were more vulnerable to anxiety and depressive symptoms, owing to greater family burden and psychological impact. The concern of carrying the virus home and passing the infection to the family was shown to be an important stress-triggering factor among healthcare workers (32).

Healthcare workers who were asked to isolate themselves for showing mild symptoms or had close contact with infected persons, also showed higher levels of anxiety and depression. Those who experienced self-isolation could fear more from being infected and worry about the health risks to their own family. This anticipation shows that much of the psychological toll was already being experienced.

The mental health of people with underlying chronic diseases has been shown to be deteriorated, which could be due to the fact that they are at a heightened risk for severe COVID-19 symptoms and increased risk of contracting the disease (33). Similarly, those with current or pre-existing psychiatric conditions also have elevated levels on both scales; which is consistent with previous studies (13). Although several factors have been shown to aggravate the psychological burden among healthcare workers, they do not seem to play a role among the severe cases. Yet, due to the small number of participants in some of the sub-groups, these results should be dealt with caution and need to be replicated in a larger sample.

Whether during the COVID-19 pandemic or before it, the prevalence of depression, anxiety and burnout has been consistently shown to be common among healthcare providers (34–36). However, the impact of the pandemic has made this worse for the healthcare professionals (37, 38). While the healthcare professionals already face the challenges of exposure risk to infection, role conflict (the professional and the family), demanding and changing work environment, uncertainty of the pandemic progress, isolation, and quarantine, the lockdown restrictions added further burden (37, 39). Mediated by social isolation, the sense of loneliness and its negative emotions can be aggravated (40, 41). This may lead to a downward vicious cycle of psychological events impacting negatively on the individual mental health including increased risk of suicide (42). On the other hand, easing the lockdown restrictions might result in improving the mental health. In the current study, we found a significant drop in the mean score of both PHQ-9 and GAD-7 after lifting of the lockdown restrictions among the healthcare workers when excluding those with a current or a pre-existing psychiatric disorder. A similar decrease in depression and anxiety was found both in the general population and healthcare workers in Wuhan, where the virus outbreak was first reported (1), after 2 months of easing the lockdown restrictions (43).

It is now obvious that the pandemic does not affect everyone equally. Regression analysis identified several predictors of anxiety and depression among healthcare workers in Saudi Arabia. Women, individuals with past or present psychiatric illnesses, nurses, individuals who experienced self-isolation, individuals living with elderly, those working in high-risk areas and individuals with physical and mental illness perception were negatively impacted.

Interestingly, these predictors varied between the two phases of the study which could be due to the rapidly changing and uncertain situation of the disease. During lockdown period, gender, psychiatric diagnosis and chronic diseases status, occupation, working areas, family infection, physical illness perception and mass screening were significant predictors for anxiety. Yet, after lifting of the lockdown, none of them remained significant. Mass active screening was also a significant predictor for anxiety during the lockdown only; however, after lifting the lockdown, this process became more structured and did not seem to have an independent effect on anxiety. After easing of lockdown restrictions, working in designated hospitals, living with elderly, and self-isolation were identified as significant predictors. In addition, participants' positive infection status and positive physical health perception could have a protective effect against anxiety. These results highlight the importance of studying the psychological impact while accounting for the phase of the pandemic. The impact as well as the people who might be negatively affected could be changed from one phase to the other. It is worth mentioning that some symptoms could be more sensitive to these changes than others. In the present study, and on the contrary to what was observed with anxiety, predicators for depression seemed to be more consistent regardless the phase of the pandemic.

The current study has several strengths. First, it measured anxiety and depression in a representative sample with adequate sample size. Second, depression and anxiety were assessed using validated scales. Third, although the cross-sectional design of the study, the assessment was done at two periods of time, during and after easing of the lockdown restrictions. Nonetheless, the study is not without limitations. The cross-sectional design and the convenient sampling method limited the causality interpretations for the decreased depression and anxiety after easing of the lockdown restrictions. Further, recruiting participants through social media could introduce a selection bias by excluding those who don't have access to these platforms. Yet, we tried to overcome this by sending the surveys using internal emails. Also, by using the online anonymous recruitment methodology, there is possibility of the respondents completing the survey more than once, but we had used a conservative approach of removing suspected duplications. There is also a possibility that some had participated in both surveys, which could affect the results. However, it would be difficult to confirm this without having personally identifiable information of the participants. In addition, self-report instruments could introduce a systematic bias in comparison to interview-based measures; but under such restrictive measures opting for the latest is challenging. Also, the application of the lockdown restrictions was not the same across different regions of the country which could have affected the mental health of the healthcare workers differently. Not including the evaluation of posttraumatic stress and/or burnout that could represent a potential confounder for anxiety and depressive symptoms is another limitation in the current study. Psychological distress/burnout has shown to contribute to the development or the persistence of psychiatric complications in healthcare workers facing COVID-19 pandemic, particularly depressive and anxiety symptoms (44). Furthermore, despite the relatively large number of participants, they were not equally distributed between regions which could hinder the generalization of these findings due to limited representation.

In light of these findings, immediate actions need to be taken to address the psychological needs of the vulnerable groups of healthcare workers as the pandemic continues. Healthcare systems should enhance strategies to face this relevant issue in healthcare workers and longer-term strategic programs should be implemented. As the situation of the pandemic changes, the rates of anxiety, depression, and other psychological issues have to be monitored. In addition, individualized tailored interventions that take into account the characteristics and socio-demographic variables of the affected individuals could be developed.

## Conclusion

The COVID-19 pandemic was associated with high rates of depression and anxiety among healthcare workers worldwide and in Saudi Arabia. Although several factors could have impacted the mental health state of the healthcare workers, but the ease of the lockdown restrictions was significantly associated with decreased mean scores of the PHQ-9 and GAD-7 among healthcare workers in Saudi Arabia. In addition, future studies with larger sample sizes would be preferable to track the trajectories of mental health outcomes among healthcare workers, in order to define mental health interventions and to design mental health care programs to deal with and minimize these psychological issues.

## Data Availability Statement

The datasets presented in this article are not readily available because they contain information that could compromise the privacy of research participants. Requests to access the datasets should be directed to Dr. Hussain M. Khrad (hkhrad@hotmail.com).

## Ethics Statement

The studies involving human participants were reviewed and approved by the local Research Advisory Committee and Research Ethics Committee of King Faisal Specialist Hospital and Research Centre-Jeddah (KFSHRC-J). The patients/participants provided their informed consent to participate in this study.

## Author Contributions

HK, WF, and AA designed the study and wrote the protocol. FB and WF performed the statistical analyses. All the authors made a significant contribution to the interpretation of the data and drafting of the manuscript, revising it critically for intellectual content, and approved the final version of the manuscript.

## Conflict of Interest

The authors declare that the research was conducted in the absence of any commercial or financial relationships that could be construed as a potential conflict of interest.

## Publisher's Note

All claims expressed in this article are solely those of the authors and do not necessarily represent those of their affiliated organizations, or those of the publisher, the editors and the reviewers. Any product that may be evaluated in this article, or claim that may be made by its manufacturer, is not guaranteed or endorsed by the publisher.
