# Supplementary material for: Lock-Down Effect on the Mental Health Status of Healthcare Workers During COVID-19 Pandemic
Source: Front Psychiatry. 2021 Aug 13;12:683603. doi: 10.3389/fpsyt.2021.683603 (PMC8414976; doi:10.3389/fpsyt.2021.683603)
Supplement: Supplementary file 1 [file Data_Sheet_1.docx]

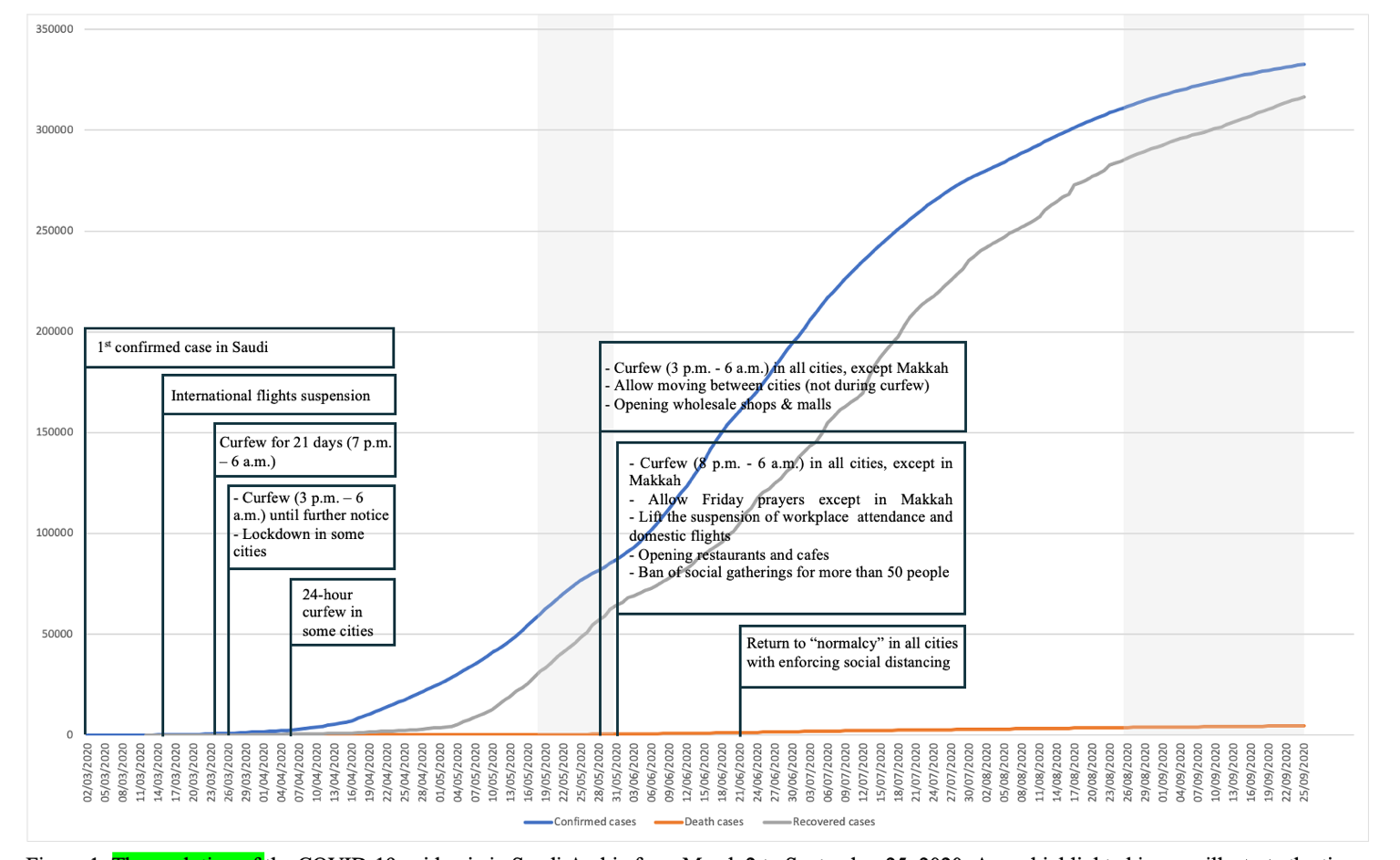


**Supplementary Figure 1.** The progression of the COVID-19 epidemic in Saudi Arabia from March 2 to September 25, 2020 and some of the early precautions and management plans which the government of Saudi Arabia had employed to curb the spread of the disease since first cases were detected. Areas highlighted in gray illustrate the times of distributing both surveys. The first survey conducted in May (between 18 -31), when curfew and lockdown were imposed. The second survey conducted between August 25 – September 25, weeks after lifting the lockdown when there was a rapid decline in the number of “daily” new cases.


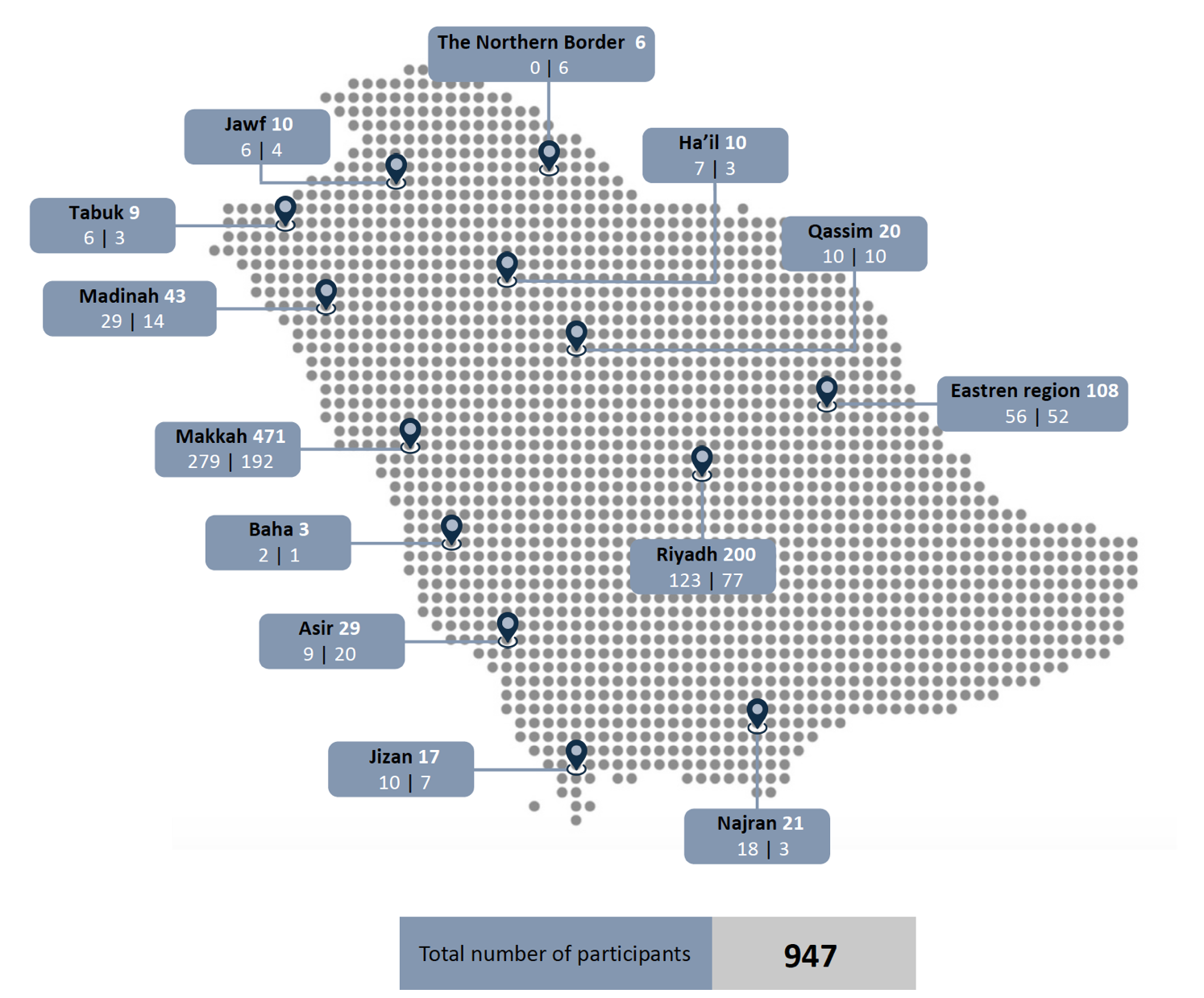


**Supplementary Figure 2.** Participants distribution over the 13 administrative regions in Saudi Arabia. Numbers on the left side indicate the number of participants in phase 1 (during the lockdown). Numbers on the right side indicate the number of participants in phase 2 (after the lockdown).
